# 

**Published:** 1915-12

**Authors:** 


### The American Society for the Study of Alcohol and Other Narcotics.

The American Society for the Study of Alcohol and Other Narcotics will hold its forty-fifth annual meeting in the parlors of the Hotel Raleigh, Washington, D. C., December 15 anad 16, 1915.

This was the first society of medical men in the world to take up the scientific study of alcohol and other narcotics. The papers and transactions have been published in the *Journal of Inebriety* and comprise the first scientific literature on the subject.

Thirty-two papers on different phases of the subject will be read at this meeting by specialists and distinguished medical and scientific men. These studies will be confined exclusively to the conclusions from laboratory observations and clinical experience.

The public is cordially invited to be present. Programs can be had by addressing the Secretary, Dr. T. D. Crothers, Hartford, Conn.

---

At a recent meeting of the Fourth District Medical Society, Brady, Texas, was selected as the next place of meeting, and Dr. J. F. Anderson, of Brady, was elected President.

